# The potential role of antimetabolite in preventing allosensitization before kidney retransplantation

**DOI:** 10.3389/fimmu.2026.1850824

**Published:** 2026-07-08

**Authors:** Madeleine Thommen, Lukas Weidmann, Dusan Harmacek, Seraina von Moos, Florian Westphal, Kerstin Hübel, Britta George, Anna Mallone, Lukas Frischknecht, Jakob Nilsson, Thomas Schachtner, Elena Rho

**Affiliations:** 1 Department of Nephrology, University Hospital of Zurich, Zurich, Switzerland; 2 Department of Nephrology, Cantonal Hospital of Lucerne, Lucerne, Switzerland; 3 University of Zurich, Zurich, Switzerland; 4 Department of Immunology, University Hospital of Zurich, Zurich, Switzerland

**Keywords:** allograft nephrectomy, allosensitization, cPRA, immunosuppression, retransplantation

## Abstract

**Background and aims:**

Retransplantation is becoming more common. Allosensitization represents an increasing issue, as it prolongs the time on the waiting list and worsens the outcome of a second transplant. The optimal maintenance immunosuppression (IS) after graft failure remains unclear.

**Methods:**

This is a retrospective single-center study including kidney retransplant recipients (KRTRs) who underwent a retransplantation in the University Hospital of Zurich between 2012 and 2023. We analyzed which factors and especially which kind of maintenance IS after graft failure could affect allosensitization.

**Results:**

In our cohort, 191/1,040 (16%) were retransplantations. Of the included KRTRs, 27/129 (20%) were living donations (LDs). The median waiting time for deceased donations (DDs) was 4 years. The burden of IS decreased from graft failure to retransplantation (triple IS 54 vs. 12%, *p* < 0.05), while the allosensitization increased [number of HLA-antibodies (Ab) MFI > 1,000/patient 5.8 vs. 12.45, *p* > 0.001]. In the univariate analysis transplant, nephrectomy was associated with a higher calculated panel reactive Ab (cPRA): 90 vs. 29 (*p* < 0.0001). At retransplantation, intake of calcineurin inhibitor (CNI) was associated with lower cPRA (27% vs. 78% no CNI, *p* < 0.001), as was intake of antimetabolite [23% for azathioprine, 20% for mycophenolic acid (MPA), and 88% for no antimetabolite, *p* < 0.001] and of prednisone (PDN) (24% vs. 66% no PDN, *p* = 0.02). First retransplantation was also associated with a lower cPRA when compared to a second or a third retransplantation (41% vs. 78% vs. 88%, *p* = 0.02). In the multivariable analysis, only the intake of antimetabolite remained associated with cPRA <85% at retransplantation. Surprisingly, we could find a weak correlation between cPRA and waiting time to retransplantation (*r* = 0.25, *R*^2^ = 0.06, *p* = 0.01).

**Conclusion:**

The intake of antimetabolite at retransplantation showed a protective effect against allosensitization. Waiting time to retransplantation had only a weak correlation with allosensitization over the whole cPRA spectrum in this Swiss cohort study.

## 1 Introduction

Kidney transplantation is the therapy of choice when it comes to renal replacement therapy (RRT) (1, 2). Still, graft function is limited in time. According to the 2023 Swiss Transplant Cohort Study (STCS) report, the kidney graft survival rates at 1, 5, and 10 years in Switzerland after deceased donation (DD) and living donation (LD) were 95%, 90%, and 84%, and 98%, 96%, and 94%, respectively. Hence, a relevant number of kidney transplant recipients (KTRs) need a second or a third transplant during their lifetime. According to the STCS report, the percentage of patients listed for a kidney retransplantation in Switzerland in 2023 was as high as 14.4% (3). Patients who require a retransplantation after graft failure have an increased risk of developing anti-human leukocyte antigen antibodies (HLA-Ab) because of the first transplantation, which induced the immune system to develop Abs against foreign HLA-antigens (4). The resulting sensitization restricts the selection of potential organ donors considerably and might lead to longer waiting times (5).

Various additional factors influence the risk and extent of allosensitization after a previous graft failure. These include the intensity and duration of immunosuppressive therapy, whether the patient had a graft nephrectomy, sensitizing events such as pregnancies or blood transfusions, and the cause of the previous graft failure (immunological vs. non-immunological) (6–8). Among these factors, immunosuppression (IS) and nephrectomy are the only modifiable ones at the time of graft failure. Hence, it is becoming increasingly relevant to understand which is the best immunosuppressive treatment for patients who experience a previous graft loss and who will be listed for a retransplantation. Practices regarding the IS management in patients with a failing graft are very heterogeneous, and guidelines on this topic are scarce and, if existent, vague about how IS should be reduced (9, 10). The Guidelines of the British Society of Transplantation about this topic, published in 2014 and updated in 2023 (11), as well as the KDIGO (Kidney Disease: Improving Global Outcomes) Executive Conclusions of Controversies Conference about management of graft decline to failure (12) agree that the decision about reduction of IS should be individualized and take into account factors such as anticipated duration of dialysis, residual kidney function, likelihood of retransplantation, degree of allosensitization, and risk of infections. Still, the above-mentioned KDIGO Executive Conclusions state that there is no agreement or sufficient evidence on how the reduction of IS should be implemented. The strategy recommended by the British Guidelines, as well as by the recommendations published on the *American Journal of Transplantation* (10), is to reduce or stop the antimetabolite at dialysis start and to continue the calcineurin inhibitor (CNI). Some studies even question whether maintaining IS reduces the risk of allosensitization (13, 14). Reducing allosensitization has traditionally been the main reason for maintaining IS, serving two key objectives: (i) maximizing the number of compatible donor organs and thereby reducing waiting times, and (ii) enhancing post-transplant outcome (15). Waiting times are, however, very much country dependent (16). This depends mainly on the organ’s availability, but also on the different allocation algorithms used in different countries. In Switzerland, the waiting time, blood group, and HLA compatibility are the main drivers of the allocation system. Priority points are assigned to recipients younger than 20 years old, to patients with a calculated panel reactive antibody (cPRA) > 98%, and to Epstein–Barr virus (EBV)-negative recipients if there is an EBV-negative donor (17). Moreover, in Switzerland, DD is possible only among recipients and donors of the same blood group, penalizing some recipients, such as those with the AB blood group.

Given the limited evidence on how to manage IS before retransplantation and the many factors that influence allosensitization and waiting time together with the rising number of retransplantation candidates and the organ shortage, a solid understanding of the contributors to allosensitization is essential. For this reason, we aimed to analyze the factors influencing allosensitization and waiting time of patients listed for a retransplantation in our Zurich cohort, with a special focus on IS.

## 2 Methods

### 2.1 Study cohort

**Figure 1:**
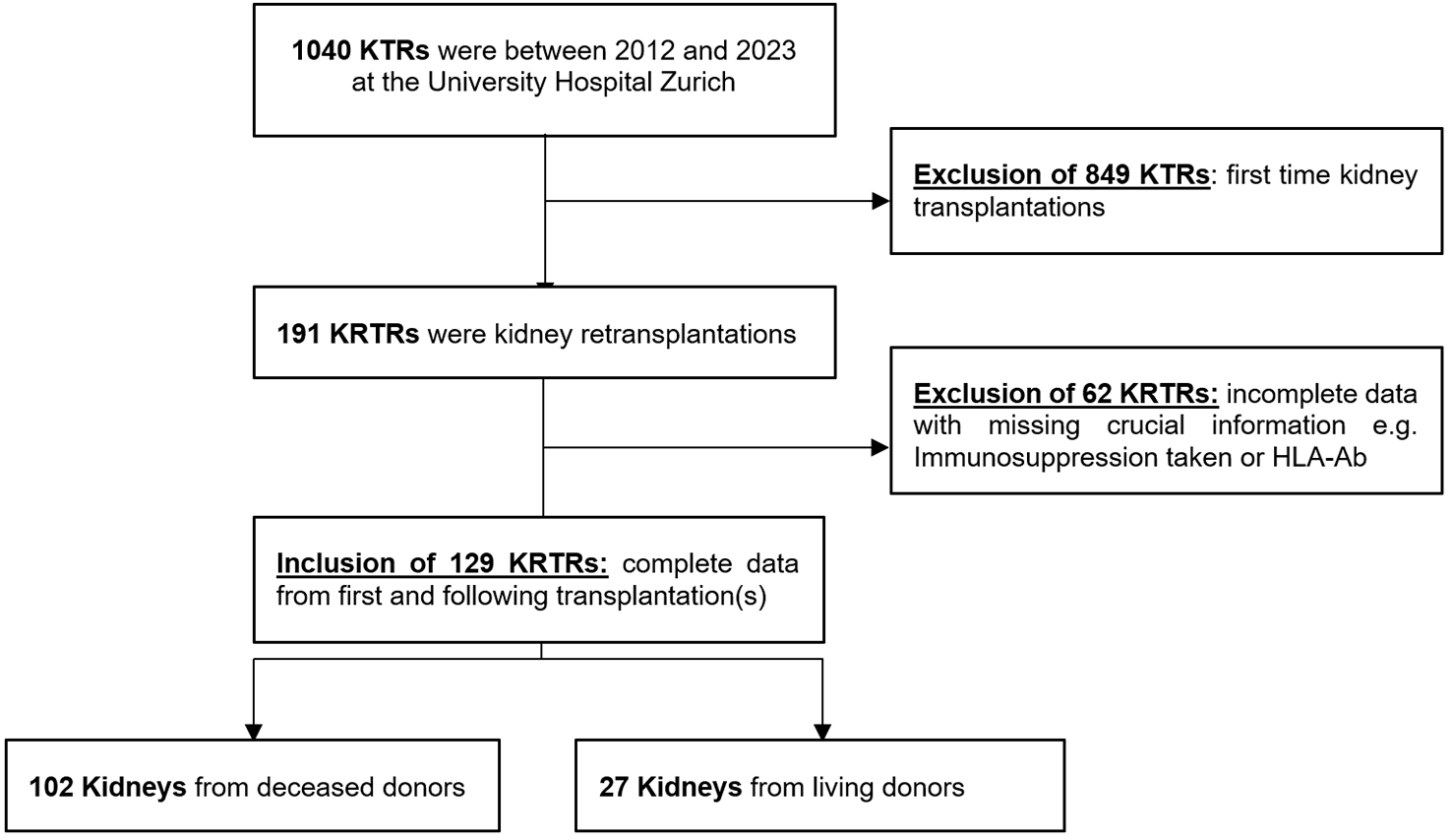
Flowchart illustrating the selection of included patients.

We conducted a retrospective single-center cohort study including KTRs who received a kidney retransplantation at the University Hospital Zurich between 2012 and 2023 after suffering a previous kidney graft failure. Data were collected by reviewing the hospital internal electronic medical records. Patients were included if data on HLA-Abs and IS were available at the time of previous graft failure, relisting, and retransplantation. The selection of patients is shown in **Figure 1**. Data were collected until 31 August 2024, which was considered the end of follow-up.

We collected demographic data and data on factors that could have an impact on allosensitization and waiting time such as kidney disease, number of retransplantation, cause of previous graft failure, duration of previous transplant, RRT modality after graft failure, nephrectomy, blood transfusions, pregnancies, and blood group.

The study was approved by the Cantonal Ethics Commission Review Board of Zurich, Switzerland (KEK-ZH-Number 2020-02817) and has been conducted in compliance with the Declaration of Helsinki.

### 2.2 Relevant time points before retransplantation

We assessed information on IS and allosensitization at three clinically relevant time points: (1) at the failing of the previous graft, (2) at listing for retransplantation, and (3) on the day of retransplantation. The time point of graft failure was defined as the last nephrological consultation in the transplant center of the University Hospital of Zurich before the establishment of RRT. This allowed the assessment of data on IS, which were not available at the start of RRT considering that many patients did not start dialysis at our center. The RRT was either hemodialysis (HD), peritoneal dialysis (PD), or pre-emptive retransplantation. Waiting time was considered from the time of dialysis start to the time of retransplantation. The analysis of waiting time and potential influencing factors was performed only with patients who received a DD.

### 2.3 Allosensitization

Allosensitization was assessed by measuring HLA-Abs class I and II, their mean fluorescence intensity (MFI), and cPRA. In particular, the number of HLA Ab of classes I and II with MFI >1,000 and >10,000 were counted at each of the above specified time points, as well as the number of patients with at least one Ab overpassing these MFI thresholds. cPRA 1,000 was defined as the percentage of donors against whom the recipients had Abs with MFI > 1,000. This MFI is the level used in Switzerland, in standard situations, to define unacceptable antigens for kidney transplantation (16). The cPRA was calculated at the time of listing and at the time of retransplantation. At the time of graft failure, only raw HLA antibody data were available, and no immunologist assessment of antibody specificity had been performed. Consequently, any calculation at this time point would not have been comparable to those at the other two time points and was therefore not carried out.

### 2.4 Immunosuppression

Since 2020, all patients at our center have received an induction therapy with anti-thymocyte globulin (ATG), except KTRs receiving an ABO incompatible transplantation, who are induced with Basiliximab and additionally received Rituximab. Dosage of ATG depends on the immunological risk stratification and on organ quality. In detail, patients at low immunological risk [i.e., without donor-specific Ab (DSA) prior to transplantation] and with donation after brain death (DBD) receive ATG in a dose of 5 mg/kg body weight (BW). Patients at high immunological risk (i.e., sensitized patients with DSA) and recipients of organs donated after cardiac death (DCD) receive ATG in a higher dose up to 21 mg/kg BW. Before 2020, standard induction was performed with Basiliximab.

Maintenance IS consists of tacrolimus (TAC) plus mycophenolic acid (MPA) either as mycophenolate mofetil (MMF) or as enteric-coated mycophenolic acid (EC-MPS) as anti-proliferative drug, with target trough levels up to months 1, 3, 6, and 12 of 10–12, 8–10, 6–8, and 5–7 ng/mL, respectively. MMF or EC-MPS is used in a dosage of 1 g or 720 mg every 12 h for patients >50 kg and 500 mg or 360 mg every 12 h for patients <50 kg. Steroids are generally reduced to 5 mg/day over the first 6 weeks after transplantation and maintained in the long term.

The management of IS after graft failure was historically delegated to the dialyzing centers without specific indication from the transplantation center on how to reduce IS.

Data about IS were also recorded at the same three time points at which allosensitization was assessed: graft failure, listing and Re-TPL.

### 2.5 Statistical methods

Statistical analyses and graphs were performed with IBM SPSS version 29.0, GraphPad Prism version 10.5, and Microsoft Excel 2016.

Continuous variables are described as mean with standard deviation if they had a normal distribution and as median with interquartile range (IQR) if they had a non-normal distribution. The displayed medians were compared using the Mann–Whitney *U* test and Kruskal–Wallis test, while means were compared with a two-sided *t*-test or analysis of variance (ANOVA), as appropriate.

Categorial variables were presented as absolute and relative frequencies and compared using the Pearson–Chi^2^ test. For categorical variables with a 2 × 2 table configuration, in which more than 20% of cells have expected frequencies <5, the Fisher exact test was performed (exact significance).

Time-related events are compared using the log-rank test (Mantel–Cox).

Correlation was performed computing Pearson correlation coefficients.

The multivariable analysis was performed for variables that biologically could have an effect on allosensitization (cPRA) and proved to have an association with outcome in the univariate analysis, which were use of CNI, use of antimetabolite, use of prednisone (PDN), previous graft nephrectomy, and having received more than one previous kidney transplantation. Odds ratios (ORs) with 95% confidence intervals (CIs, low and high) for cPRA were calculated using binary logistic regression models, using as outcome a cPRA > 85%.

The multivariate analysis for waiting time was performed for variables that proved to have an association in the univariate analysis, which were CNI, antimetabolite, blood group, and nephrectomy.

A two-sided *p*-value <0.05 was assumed as statistical significance for all tests.

## 3 Results

### 3.1 Patient characteristics

Out of 1,040 KTRs between 2012 and 2023, 191 (16%) were kidney retransplant recipients (KRTRs). These 191 KRTRs were considered for inclusion into this study. A total of 62 KRTRs were missing information regarding the management of IS before retransplantation and were therefore excluded. Hence, the final cohort for analysis consisted of 129 KRTRs (**Figure 1**).

Baseline characteristics of patients are shown in **Table 1**.

**Table 1:** Baseline characteristics of 129 included KTRs.

	Deceased donor	Living donor	Total group	p
	102	27	129	
Recipient characteristics				
Age at TPL*	53 (43.0–60.75)	44 (40.50–56.0)	52 (42.0–60.0)	0.104
Men, n (%)	71 (69%)	9 (33%)	80 (62%)	0.001
Blood group, n (%)				0.5
A	56 (54.90%)	12 (44.44%)	68 (52.71%)	
B	7 (6.86%)	1 (3.70%)	8 (6.20%)	
O	36 (35.29%)	12 (44.44%)	48 (37.21%)	
AB	3 (2.94%)	2 (7.41%)	5 (3.88%)	
Cause of previous TPL-loss, n (%)				0.214
Rejection	38 (37.25%)	9 (33.33%)	47 (36.43%)	
BK-nephropathy	3 (2.94%)	3 (11.11%)	6 (4.65%)	
CNI-toxicity	5 (4.90%)	0	5 (3.88%)	
Hypertensive nephropathy	1 (0.98%)	1 (3.70%)	2 (1.55%)	
Primary non-function	4 (3.92%)	0	4 (3.10%)	
Recidive/de novo glomerulonephritis	13 (12.75%)	5 (18.52%)	18 (13.95%)	
Graft-thrombosis	2 (1.96%)	0	2 (1.55%)	
Unknown	34 (33.33%)	8 (29.63%)	42 (32.56%)	
Urologic complications	0	1 (3.70%)	1 (0.78%)	
RRT after graft-loss, n (%)				0.014
HD	83 (81.37%)	19 (70.37%)	102 (79.07%)	
PD	5 (4.90%)	2 (7.41%)	16 (12.40%)	
Preemptive retransplantation	14 (13.73%)	6 (22.22%)	11 (8.53%)	
Time in years				
Waiting time from RRT*	4.03 (2.4–5.6)	0.98 (0.45–2.20)	3.12 (1.43–5.25)	<0.01
Waiting time from listing*	3.23 (1.85–5–06)	1.11 (0.67–3.96)	2.84 (1.18–4.42)	<0.01
Potentially sensitizing events				
Blood transfusion, n (%)	58 (57%)	11 (41%)	69 (58%)	0.19
Pregnancy (only women), n (standard deviation)	0.35 (±0.661)	0.72 (±0.826)	0.49 (±0.739)	0.019
Nephrectomy, n (%)	28 (27.45%)	7 (25.93%)	35 (27.13%)	0.957
No. of KTPL				
Second Third Fourth	88 (86%) 13 (13%) 1 (1%)	24 (89%) 2 (7%) 1 (4%)	112 (87%) 15 (12%) 2 (2%)	0.48
Duration of previous graft* in years	10.36 (3.85–16.5)	9.69 (2.81–17.14)	10.33 (3.87–15.95)	0.38

*Median (interquartile range).

KRTRs had a median age of 52 (IQR 42–60), 80/129 (62%) were male, 102/129 (79.1%) received a DD, and 27/129 (20.9%) received an LD. A total of 112/129 (86.8%) received a second transplant, 15/129 (11.6%) had a third transplant, and 2/129 (1.6%) received a fourth transplant. Function of previous graft in the total cohort was 10.33 (IQR 3.87–15.95) years. Blood group distributions were as follows: A: 52.7%; B: 6.2%; O: 37.2%; AB: 3.9%. The most common reason for previous graft failure was rejection in 47/129 (36.4%) KRTRs. Four out of 129 (3%) patients suffered a primary non-function of the previous graft. Sensitizing events such as blood transfusions, nephrectomies, and pregnancies were recorded in 69/129 (53%), 35/129 (27.1%), and 17/49 (35% of women) KRTRs, respectively. In a relevant number of KRTRs, 49/129 (38%), transfusion status was unknown. Previous pregnancies were more frequent in women who received an LD (72% vs. 35%, *p* = 0.019).

### 3.2 Immunosuppression-related characteristics

**Figure 2:**
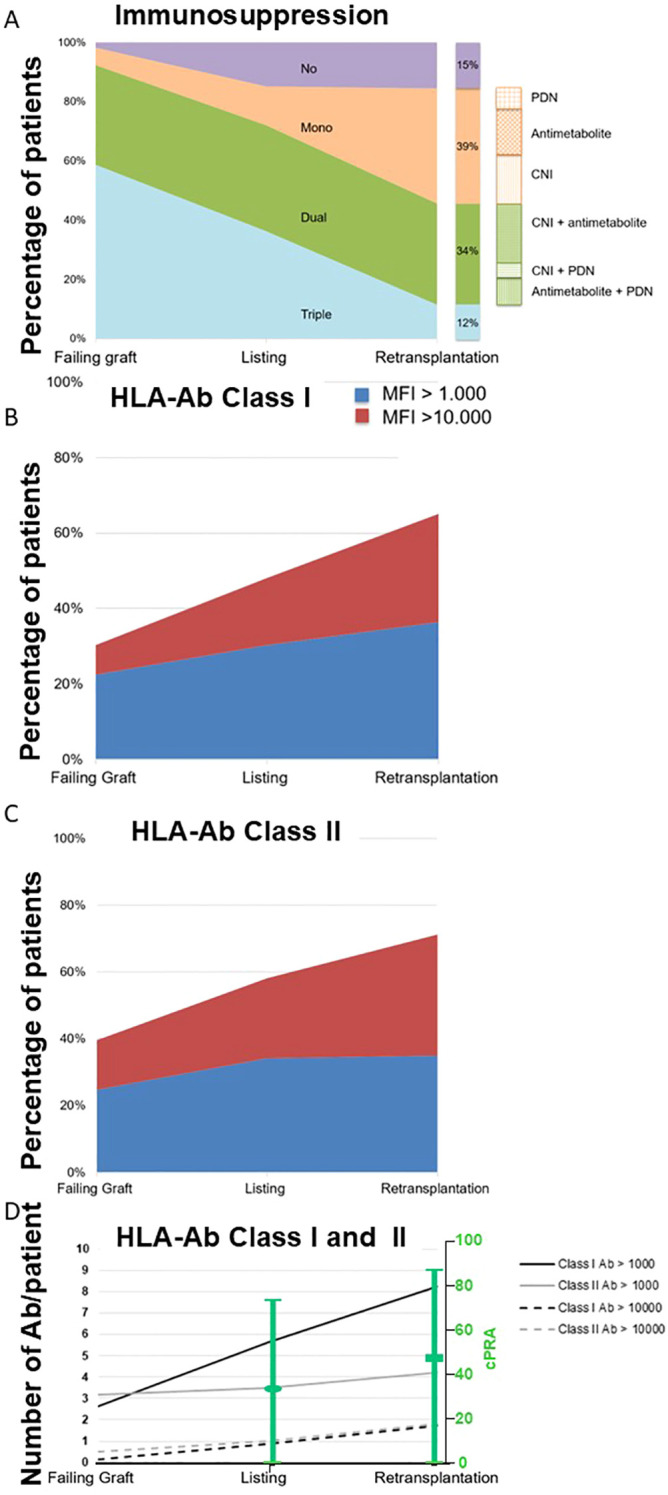
Burden of immunosuppression and allosensitization from failing graft to retransplantation. In each graph are depicted on the *x* axis the three time points: failing graft, listing for retransplantation, and retransplantation itself. In graph **(A)**, the number of used immunosuppressants is represented over these three time points; in graph **(B)**, the percentage of patients with HLA Class I antibody is shown; in graph **(C)**, the percentage of patients with Class II antibodies is denoted; and in graph **(D)**, the mean of antibodies per patient and cPRA at listing and retransplantation are indicated.

IS-related characteristics are shown in **Table 2** and **Figure 2**. At the time of graft failure, 70/129 (54%) patients were taking triple IS while 40/129 (31%) were taking dual IS and 7/129 (5.5%) were on mono IS. A total of 12/129 (9%) were not taking any IS at this time. At the time point of listing, 47/129 (36%) were on triple IS, 46/129 (36%) were on dual IS, and 17/129 (13%) were on mono IS, while 19/129 (14%) were without IS. On the day of retransplantation, 15/129 (12%) were on triple IS, 44/129 (34%) were on dual IS, 50/129 (39%) were on mono IS, while 20/129 (15%) were not taking any IS. Hence, the number of patients with triple IS significantly decreased from graft failure to retransplantation (54% vs. 12%, *p* < 0.05), while the number of mono IS significantly increased (5% vs. 39%, *p* < 0.05). The proportion of patients with dual IS remained similar (31% vs. 34%, *p* = 0.724). There were different combinations of dual IS with a predominance of CNI in combination with antimetabolite. The use of CNI + PDN was similar at the three time points: (7% vs. 5% vs. 5%, *p* = 0.146); while the use of CNI + antimetabolite (21% vs. 26% vs. 20%, *p* = 0.042) was less frequent at listing and the use of antimetabolite + PDN increased over time (3% vs. 5% vs. 9%, *p* ≤ 0.05). In case of the mono IS, there was a continuous increase over the three measurement points, even if this increase for CNI and antimetabolite monotherapy was not statistically significant: CNI (2% vs. 6% vs. 16%, *p* = 0.176), antimetabolite (2% vs. 3% vs. 15%, *p* = 0.556), and PDN (2% vs. 4% vs. 7%, *p* = 0.050).

**Table 2:** Immunosuppression-related characteristics.

	Failing graft	Listing for retransplantation	Retransplantation	p
Immunuppression, n (%)				< 0.05
Triple IS	70 (54.26%)	47 (36.4%)	15 (11.6%)	
Dual	40 (31.01%)	46 (35.7%)	44 (34.1%)	
CNI with PDN	9 (6.98%)	7 (5.4%)	6 (4.7%)	
CNI with antimetabolite	27 (20.93%)	33 (25.6%)	26 (20.16%)	
Antimetabolite with PDN	4 (3.10%)	6 (4.7%)	12 (9.3%)	
Mono IS	7 (5.43%)	17 (13.2%)	50 (38.8%)	
CNI	2 (1.55%)	8 (6.20%)	21 (16.28%)	
Antimetabolite	2 (1.55%)	4 (3.10%)	20 (15.5%)	
PDN	3 (2.33%)	5 (3.88%)	9 (6.98%)	
None	12 (9.3%)	19 (14.73%)	20 (15.5%)	¨

CNI use is shown in **Supplementary Figure 1**. CNI trough levels were measured at the two time points of failing graft and retransplantation. At the time of failing graft, 71/129 (55.0%) patients received TAC and 35/129 (27.1%) received Ciclosporin (CsA). Among the TAC users, the levels were <5 ng/mL in 23/71 (17.8%) patients, between 5 and 7 ng/mL in 7/71 (13.2%) patients, and >7 ng/mL in 16/71 (23.0%) patients; for 24 patients, no data were available. CsA was taken by 35/129 (27.1%) patients: 9/35 (26.0%) patients, <50 ng/mL; 4/35 (11%) patients, 50 to 80 ng/mL; and 1/35 (3%) patients, >80 ng/mL. For 21 (60%) patients, no CsA level was available. At the time of retransplantation, 51/129 (39.5%) patients were under TAC and 17/129 (13.2%) patients received CsA. In the patients taking TAC, 5/51 (10%) had a trough level <5 ng/mL and 4/35 (8%) had a trough level >7 ng/mL; for the remaining 42 (82%) patients, no data were available. For the vast majority of KTRs under CsA, there were no data available on the through level on the day of retransplantation. The most prescribed dose of MMF at the time of retransplantation was 1,000 mg/day, which was prescribed in 32/66 (48%) of the KRTRs who were still taking this drug, with a mean dose of 763 ( ± 331) mg/day.

### 3.3 Allosensitization-related characteristics

Allosensitization-related characteristics are shown in **Table 3** and **Figure 2**.

**Table 3:** Allosensitization-related characteristics.

	Failing graft	Listing for retransplantation	Retransplantation	p
Allosensitization, *n* (%)				
No. of patients with HLA-Class I Ab with MFI > 1,000	39 (30.23%)	62 (48.06%)	84 (65.12%)	0.649
No. of patients with HLA-Class II Ab with MFI > 1,000	51 (39.53%)	75 (58.14%)	92 (71.3%)	0.012
No. of patients with HLA-Class I Ab with MFI > 10,000 (AVOIDS)	10 (7.75%)	23 (17.83%)	37 (28.7%)	<0.001
No. of patients with HLA-Class II Ab with MFI > 10,000 (AVOIDS)	19 (14.73%)	31 (24.03%)	41 (36.4%)	0.074
Mean no. of antibodies/patients (SD) according to Class and MFI				
HLA-Class I Ab with MFI > 1,000 per patient	2.64 (±5.47)	5.62 (±10.51)	8.24 (±11.55)	<0.001
HLA-Class II Ab with MFI > 1,000 per patient	3.16 (±5.74)	3.51 (±5.54)	4.21 (±5.46)	0.629
HLA-Class I Ab with MFI > 10,000 (AVOIDS) per patient	0.14 (±0.54)	0.87 (±2.86)	1.74 (±4.93)	0.001
HLA-Class II Ab with MFI > 10,000 (AVOIDS) per patient	0.53 (±1.27)	1.02 (±2.28)	1.84 (±3.88)	0.001
cPRA per patient	–	33.29 (0–73)	46.99 (0.15–86)	0.02

At the time point of graft failure, 39/129 (30%) KRTRs had at least one Class I HLA Ab with MFI > 1,000 and 10/129 (8%) had an MFI > 10,000. At the time point listing, 62/129 (50%) KRTRs had Class I HLA Ab with MFI > 1,000 and 23/129 (18%) had an MFI > 10,000. At the time point retransplantation, 84/129 (65%) KRTRs had Ab Class I HLA with MFI > 1,000 and 37/129 (29%) had an MFI > 10,000. The increase in number of sensitized patients was significant for MFI > 1,000 (47% vs. 70% vs. 86%, *p* < 0.001), as well as for MFI > 10,000 (8% vs. 18% vs. 29%, *p* < 0.001). Similar observations were true for KRTRs with Class II HLA Ab with a significant increase in number of sensitized patients for MFI > 1,000 (40% vs. 58% vs. 71%, *p* = 0.012) as well as for number of sensitized patients with Ab with MFI > 10,000 (15% vs. 24% vs. 36%, *p* = 0.07).

At listing, the median cPRA was 33.29 (0–73), while at the day of retransplantation, the median cPRA was 47 (0.15–86), as depicted in **Figure 2D**.

### 3.4 Waiting time

The median waiting time from graft failure to retransplantation was 3.13 (IQR 1.43–5.25) years with a clear difference between DD and LD (4.03 years vs. 0.93 years, *p* < 0.01).

**Figure 3:**
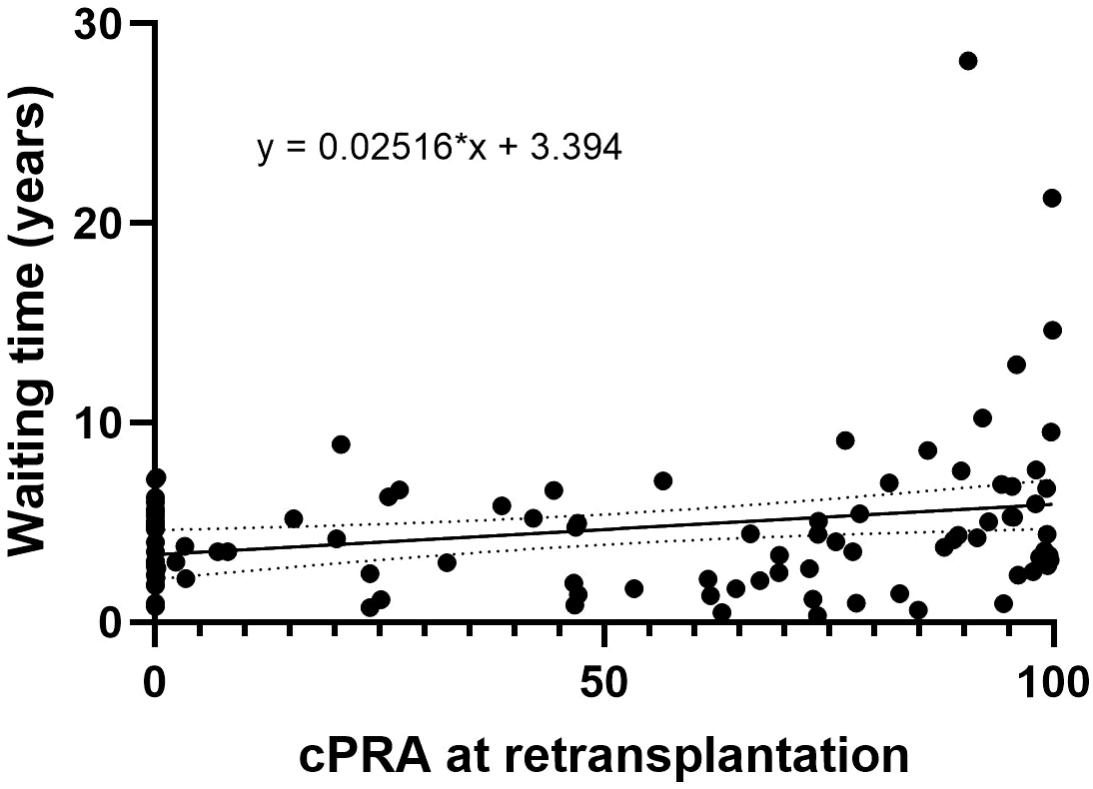
Correlation between waiting time in years for retransplantation on the *y* axis and allosensitization on the *x* axis expressed as cPRA at the time of retransplantation. cPRA (0%–100%) represents the estimated percentage of donors against whom a recipient has preformed anti-HLA antibodies; higher values indicate greater sensitization.

Interestingly, in our cohort, we could observe only a weak correlation between cPRA at the time of retransplantation and waiting time after DD (*r* = 0.25, *R*^2^ = 0.06, *p* = 0.01) as shown in **Figure 3**. Still, a clinically relevant difference in waiting time was observed for higher cPRA. Hence, the median waiting time for KRTR with cPRA > 90 was 5.3 (IQR 3.2–8.1) years, as compared to 3.8 (IQR 2–5.4) KRTR with cPRA < 90 (*p* = 0.02), even if the candidates with a cPRA > 98 were prioritized.

### 3.5 Factors correlating with allosensitization

**Figure 4:**
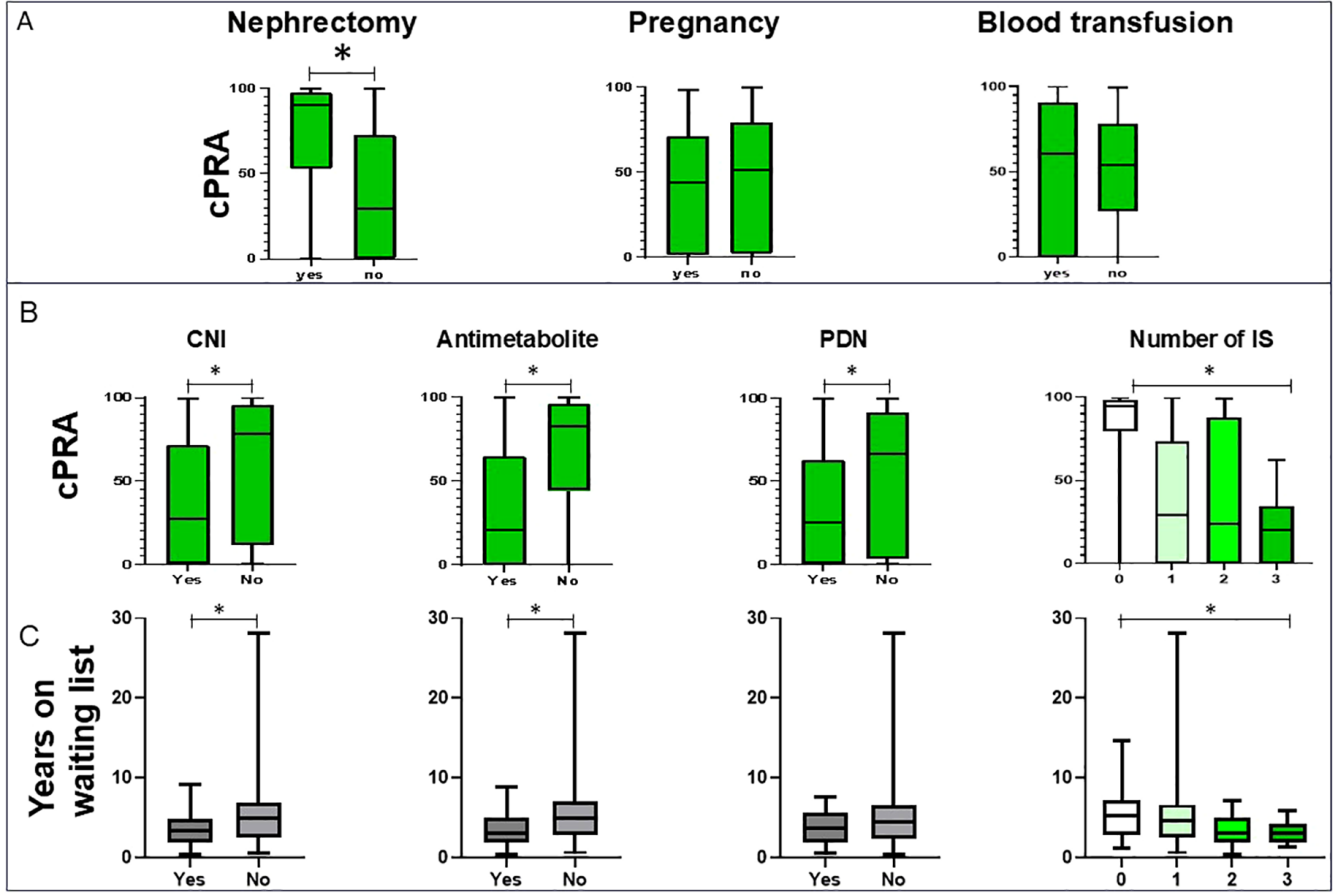
Effect of sensitizing events such as nephrectomy, pregnancy and blood transfusions on cPRA **(A)**. Effect of immunosuppression on cPRA **(B)** and waiting time **(C)**. The asterisk (*) indicates statistical significance (p < 0.05).

In the univariate analysis, patients with more than one previous transplants had significantly higher median cPRA than those with only one previous transplant 78.98 (IQR 69.43–94.67) vs. 41.47 (IQR 0.05–83.35) (*p* = 0.006). Undergoing dialysis prior to retransplantation was associated with a median cPRA of 59.02 (1.56–89.26) vs. 0.77 (IQR 0–29.58) in patients without RRT (*p* = 0.009). The same was true for previous nephrectomy, which was associated with a higher cPRA of 90.46 (IQR 52.78–97.65) vs. 29.30 (IQR 0–73.00) (*p* < 0.001), as shown in **Figure 4**. A waiting time of more than >5 years was also associated with a higher cPRA, 80.07 (IQR 24.66–95.61) vs. 47.04 (IQR 0.02–77.17) (*p* = 0.01). There was no significant association between cPRA and gender (*p* = 0.524). Likewise, cPRA did not differ between patients with and those without previous pregnancies—43.71 (IQR 1.31–70.92) vs. 51.20 (IQR 2.33–79.31), *p* = 0.46. No significant difference in cPRA was observed according to blood transfusion status, with median values of 61 (IQR 0.1–90.1) in transfused patients and 38.59 (IQR 30.6–77) in non-transfused patients (*p* = 0.33).

The effect of IS on allosensitization is shown in **Figure 4** and **Supplementary Figure 2**. In the univariate analysis, use of CNI was associated with lower cPRA (27.21 vs. 78.01, *p* < 0.001); this was also true for use of antimetabolites at the time of retransplantation (20.68 vs. 82.84 *p* ≤ 0.0001) and of PDN (24.94 vs. 66.80 *p* = 0.02). The use of a higher number of IS was also associated with lower cPRA: 2.33 (IQR: 0–34) for three medications; 20 (IQR: 0–65) for two medications, 69 (IQR: 20–92) for mono IS, and 84 (IQR: 49–96) for none, *p* < 0.001.

In the multivariable analysis, a cPRA > 85% was negatively associated with the intake of antimetabolite at retransplantation (OR 0.2, CI 0.07–0.57, *p* = 0.003). The intake of CNI (OR 0.39, CI 0.14–1.1, *p* = 0.07) and PDN (0.86, CI 0.3–2.5, *p* = 0.78) as well as a previous nephrectomy (2.78, CI 0.90–7.89, *p* = 0.07) or being retransplanted more than once (0.69, CI 0.19–2.56, *p* = 0.58) were not statistically associated with a higher risk of cPRA >85% in the multivariable analysis.

To better understand the relative protective role provided by the different kinds of immunosuppressants against allosensitization, we performed some additional analysis. cPRA was compared for KRTR under mono IS: CNI users (*n* = 21) had a median cPRA of 67 (IQR 22–75), antimetabolite users (*n* = 20) had a median cPRA of 50 (IQR 0–67), and steroid users (*n* = 9) had a median cPRA of 13 (IQR 0–81), p = 0.34. Moreover, cPRA was compared among CNI-based dual IS (e.g., CNI+steroids, *n* = 6) with a median cPRA of 47 (IQR 1.7–89.3) and antimetabolite-based dual IS (e.g., antimetabolite+steroids, *n* = 12) with a median cPRA of 13 (IQR 0–69), *p* = 0.39.

### 3.6 Factors correlating with waiting time

In the univariate analysis, longer waiting times could be observed depending on the number of previous transplants [one vs. several; 3.7 (IQR 2.3–5.3) years vs. 5.5 (IQR 3.4–10.8) years, *p* = 0.02]. The median waiting time differed according to blood group: A: 3.12 (IQR 1.99–5.21) years, B: 4.14 (IQR 1.69–7.09) years, AB: 6.02 (IQR 6.02–28.14) years, and O: 5.01 (IQR 3.29–6.53) years (*p* = 0.044). A tendency for longer waiting times could be observed for patients who received blood transfusion [4.3 years (IQR 2.4–5.7) vs. 2.4 (IQR 1.8–4.2)], but even in this case, the difference was statistically not significant (*p* = 0.12). Female KRTRs who had a previous pregnancy did not wait longer than those who did not (3.9 vs. 3.8 years, *p* = 0.99).

The effect of IS on waiting time is shown in **Figure 4**. A lower number of immunosuppressants at the time of retransplantation correlated with longer waiting times with respectively 5.23 (IQR 2.8–7.3), 4.6 (IQR 2.5–6.6), 3.1 (IQR 1.9–5.1), and 3 (IQR 1.9–4.2) years for 0, 1, 2, and 3 immunosuppressants (*p* = 0.02).

The intake of CNI was associated with shorter waiting times (3.37 vs. 4.98 years, *p* = 0.005), as well as the intake of an antimetabolite (3.1 vs. 4.9 years, *p* = 0.001). This was not the case for the intake of PDN (3.6 vs. 4.4 years *p* = 0.34). We renounced to perform further subgroup analysis among KRTRs who were taking mono or dual IS because of the very small sample sizes of the subgroups limiting the meaningfulness of such analysis.

In the multivariable linear regression, CNI and antimetabolite retained a similar effect size but did not reach statistical significance (CNI: *B* = −2.16 years, *p* = 0.081; antimetabolites: *B* = −2.28 years, *p* = 0.062), suggesting a trend toward shorter waiting times.

## 4 Discussion

In our cohort, 16% of transplanted patients underwent a retransplantation, data that are in line with publications from other countries (18, 19). As expected, the median waiting time for a retransplantation from a DD was longer than the median national waiting time with 4 as compared to 2.6 years, respectively (3). The longest waiting time was observed for KRTRs with a cPRA >90% with a median waiting time of 5.3 years.

Previous publications from Austria (20) and Oceania (21) showed that longer waiting times before retransplantation are associated with poorer outcomes in terms of patient and graft survival, with the survival benefit associated with transplantation being not statistically relevant after a waiting time of 3 years and vanishing completely when patients are waiting longer than 8 years (20). Similar findings were also found in previous studies of our center and other transplantation centers, showing that longer times on dialysis before retransplantation correlated with a worse graft survival (22, 23). It is therefore of paramount importance to aim for pre-emptive transplantation and to be informed about waiting times at the referral transplantation center, as well as any modifiable factors that could help shorten the waiting time (24).

Only a weak correlation between waiting time and allosensitization expressed as cPRA at the time of retransplantation could be shown. Still, a longer waiting time was observed for KRTRs with a higher cPRA, so that this should also be monitored and considered when adjusting IS before retransplantation. Importantly, the weak correlation implies that it is not essential to aim for a cPRA of 0, possibly at the price of a higher infection rate, but rather to avoid an extreme sensitization.

We could show that IS has a certain effect on cPRA, especially the idea that antimetabolite was associated with a lower cPRA at retransplantation. Recent studies investigating the optimal immunosuppressive strategy to prevent allosensitization after graft failure (5, 8, 25, 26) have focused mainly on the role of CNIs rather than antimetabolite. This is likely because current guidelines and expert opinions (10–12) most commonly recommend CNI-based strategies. These studies suggest that longer exposure to CNIs is associated with lower sensitization, at the cost of acceptable infection rates (6). Our KRTRs were treated mainly in external dialysis centers, and historically, the management of IS after graft failure has been delegated to the external nephrologists, explaining the heterogeneity of strategies that were used and possibly leading to confounding by indication. Still, this heterogeneity allowed for some interesting observations. Our findings confirm that continued exposure to CNI was associated with a lower risk of sensitization, but notably, in our multivariable analysis, the protective effect of antimetabolite therapy—predominantly MMF, which was used by the vast majority of our patients—appeared even stronger than that observed for CNIs. A more robust analysis assessing the role of the relative contributions of the single immunosuppressants was unfortunately not conclusive because of the small sample sizes of the subgroups. Still, these findings suggest a potentially underappreciated role of MMF in preventing allosensitization after graft failure and warrant further investigation in prospective studies specifically designed to assess the relative contribution of different immunosuppressive agents. To our knowledge, this is the first retrospective cohort study to highlight the importance of antimetabolites in preventing allosensitization after graft failure.

Second, we found that higher levels of IS at the time of retransplantation did not prevent an increase in HLA Class II Abs, which are important not only for identifying a compatible organ but also for post-transplant outcomes. IS, especially if less intense, prevents antibody formation incompletely and Class II escapes more often). This might be because traditional IS strongly blocks T-cell activation, but incompletely blocks T follicular helper germinal center response, on which germinal center and memory B cells depend to produce Class II Abs (27).

The lack of effect of IS on HLA Class II Abs may be related to the reduced maintenance doses used after the first graft failure. In our cohort, we had reliable data on antimetabolite dosing, with MMF 1,000 mg/day being the most commonly prescribed dose at the time of retransplantation. This dose can achieve area-under-the-curve values within the recommended therapeutic range in a substantial proportion of KTRs. In contrast, following graft failure, CNI trough targets are often intentionally low—typically 2–4 µg/L for TAC or 20–40 µg/L for CsA. This may explain why, in the multivariable analysis, the effect of CNIs was no longer evident compared with MMF in preventing allosensitization. Lucisano et al. (8) showed that a TAC trough level >3 µg/L should be targeted to prevent significant sensitization. In our cohort, trough levels were available for only a small subset of patients, and some values suggested that they were not true trough measurements; therefore, this explanation remains speculative.

Third, our study confirms previous findings (9, 28, 29) that identify allograft nephrectomy as a clear deleterious factor for allosensitization. While this association did not achieve statistical significance in the multivariable analysis in our study, the available literature supports limiting allograft nephrectomy to highly selected clinical indications. It is indeed becoming more and more evident that nephrectomy *per se*, is associated to higher sensitization, and not necessarily the reduction of IS, which is usually associated to it (6). Different studies tried to explain this aspect, and a possible plausible explanation is the so-called “sponge effect”, which describes the phenomenon of Abs being not detectable in the bloodstream as long as they are confined in the graft exposing the antigens against which they are directed to. This “sponge effect” vanishes after graft-nephrectomy and therefore the circulating HLA-Abs increase after it (30).

Although relevant for the clinical practice, our findings have to be interpreted in light of some important limitations. Firstly, we considered only the patients who got a retransplantation and not all those who were listed for a retransplantation, possibly missing those who were waiting even longer or never got transplanted (31) and introducing an immortality bias. This important limitation could fortunately not be avoided because we did not have the data of listed patients, which get managed on a national level. This limits the conclusions of our study and rather than providing definitive evidence, our work should be viewed as hypothesis-generating. Secondly, because the vast majority of these patients were dialyzing in external dialysis centers, we had no data about infections that occurred during the waiting time, which is always an important aspect to consider when adjusting IS after a previous graft failure (32). Another limitation is that allosensitization at graft failure was assessed only with the raw data coming from the One Lambda Luminex analysis, without a more critical interpretation of the HLA-Abs, which was not possible for the amount of data we considered.

Nevertheless, our study provides clinically relevant insights. It confirms that nephrectomy should be avoided whenever possible and suggests that the role of MMF warrants further investigation in prospective trials, ideally in direct comparison with CNIs, to determine the most effective immunosuppressive strategy for preventing allosensitization after transplant failure.

## Data Availability

The original contributions presented in the study are included in the article/**Supplementary Material**. Further inquiries can be directed to the corresponding author.

## Supplementary material

Supplementary Figure 1 CNI levels at the time point of failing graft and the day of retransplantation.

Supplementary Figure 2 Effect of immunosuppression on HLA antibodies. **(A)** Number of Class I Antibodies with MFI > 1,000 per patient at time of retransplantation. **(B)** Number of Class I Antibodies with MFI > 10,000 per patient at time of retransplantation. **(C)** Number of Class II Antibodies with MFI > 1,000 per patient at time of retransplantation. **(D)** Number of Class II Antibodies with MFI > 10,000 per patient at time of retransplantation.

## References

[1] Chaudhry D Chaudhry A Peracha J Sharif A . Survival for waitlisted kidney failure patients receiving transplantation versus remaining on waiting list: systematic review and meta-analysis. Bmj. (2022) 376:e068769. doi: 10.1136/bmj-2021-068769 35232772 PMC8886447

[2] Abecassis M Bartlett ST Collins AJ Davis CL Delmonico FL Friedewald JJ . Kidney transplantation as primary therapy for end-stage renal disease: a National Kidney Foundation/Kidney Disease Outcomes Quality Initiative (NKF/KDOQITM) conference. Clin J Am Soc Nephrol. (2008) 3:471–80. doi: 10.2215/cjn.05021107 18256371 PMC2390948

[3] Study STC . Swiss Transplant Cohort Study Report (January 2008 - 31 December 2024) (2025). Available online at: https://wwwsfndtorg/sites/wwwsfndtorg/files/medias/documents/SFNDT_guide%20complet-VF-HDpdf (Accessed February 28, 2026).

[4] Alelign T Ahmed MM Bobosha K Tadesse Y Howe R Petros B . Kidney transplantation: the challenge of human leukocyte antigen and its therapeutic strategies. J Immunol Res. (2018) 2018:5986740. doi: 10.1155/2018/5986740 29693023 PMC5859822

[5] Allesina A Lavacca A Fop F Giraudi R Giovinazzo G Deaglio S . Significant long-term prevention of high sensitization after kidney allograft failure by maintaining calcineurin inhibitor-based immunosuppression. Clin Transplant. (2024) 38:e15394. doi: 10.1111/ctr.15394 39001595

[6] Garg N Viney K Burger J Hidalgo L Parajuli S Aziz F . Factors affecting sensitization following kidney allograft failure. Clin Transplant. (2022) 36:e14558. doi: 10.1111/ctr.14558 34923658

[7] Pandey P Pande A Mandal S Devra AK Sinha VK Bhatt AP . Effects of different sensitization events on HLA alloimmunization in renal transplant cases; a retrospective observation in 1066 cases. Transpl Immunol. (2022) 75:101680. doi: 10.1016/j.trim.2022.101680 35908630

[8] Lucisano G Brookes P Santos-Nunez E Firmin N Gunby N Hassan S . Allosensitization after transplant failure: the role of graft nephrectomy and immunosuppression - a retrospective study. Transpl Int. (2019) 32:949–59. doi: 10.1111/tri.13442 30980556

[9] Davis S Mohan S . Managing patients with failing kidney allograft: many questions remain. Clin J Am Soc Nephrol. (2022) 17:444–51. doi: 10.2215/CJN.14620920 PMC8975040 33692118

[10] Lubetzky M Tantisattamo E Molnar MZ Lentine KL Basu A Parsons RF . The failing kidney allograft: a review and recommendations for the care and management of a complex group of patients. Am J Transplant. (2021) 21:2937–49. doi: 10.1111/ajt.16717 34115439

[11] Society BT . UK Guideline for the Management of the Patient With a Failing Kidney Transplant (2023). Available online at: https://btsorguk/uk-guideline-for-the-management-of-the-patient-with-a-failing-kidney-transplant/ (Accessed February 28, 2026).

[12] Josephson MA Becker Y Budde K Kasiske BL Kiberd BA Loupy A . Challenges in the management of the kidney allograft: from decline to failure: conclusions from a Kidney Disease: Improving Global Outcomes (KDIGO) Controversies Conference. Kidney Int. (2023) 104:1076–91. doi: 10.1016/j.kint.2023.05.010 37236423

[13] Martin K Cantwell L Barraclough KA Lian M Masterson R Hughes PD . Prolonged immunosuppression does not improve risk of sensitization or likelihood of retransplantation after kidney transplant graft failure. Transpl Int. (2021) 34:2353–62. doi: 10.1111/tri.13998 34320262

[14] Elgenidy A Shemies RS Atef M Awad AK El-Leithy HH Helmy M . Revisiting maintenance immunosuppression in patients with renal transplant failure: early weaning of immunosuppression versus prolonged maintenance-systematic review and meta-analysis. J Nephrol. (2023) 36:537–50. doi: 10.1007/s40620-022-01458-y 36109426

[15] de Rougemont O Deng Y Frischknecht L Wehmeier C Villard J Ferrari-Lacraz S . Donation type and the effect of pre-transplant donor specific antibodies - Data from the Swiss Transplant Cohort Study. Front Immunol. (2023) 14:1104371. doi: 10.3389/fimmu.2023.1104371 38361942 PMC10867099

[16] Bertacchi M Ferrari-Lacraz S Nilsson J Thaqi A Schmutz Y Wehmeier C . Assessment of access and outcomes of kidney transplantation through the reforms of the Swiss organ allocation system. Front Public Health. (2024) 12:1500781. doi: 10.3389/fpubh.2024.1500781 39845654 PMC11751015

[17] Bundesrat S . Verordnung Über Die Zuteilung Von Organen Zur Transplantation (Organzuteilungsverordnung) (2007). Available online at: https://wwwfedlexadminch/eli/cc/2007/281/de (Accessed February 28, 2026).

[18] Schold JD Augustine JJ Huml AM O'Toole J Sedor JR Poggio ED . Modest rates and wide variation in timely access to repeat kidney transplantation in the United States. Am J Transplant. (2020) 20:769–78. doi: 10.1111/ajt.15646 31599065 PMC7204603

[19] Heaphy EL Poggio ED Flechner SM Goldfarb DA Askar M Fatica R . Risk factors for retransplant kidney recipients: relisting and outcomes from patients' primary transplant. Am J Transplant. (2014) 14:1356–67. doi: 10.1111/ajt.12690 24731101

[20] Kainz A Kammer M Reindl-Schwaighofer R Strohmaier S Petr V Viklicky O . Waiting time for second kidney transplantation and mortality. Clin J Am Soc Nephrol. (2022) 17:90–7. doi: 10.2215/cjn.07620621 34965955 PMC8763155

[21] Wong G Chua S Chadban SJ Clayton P Pilmore H Hughes PD . Waiting time between failure of first graft and second kidney transplant and graft and patient survival. Transplantation. (2016) 100:1767–75. doi: 10.1097/tp.0000000000000953 26457605

[22] Ehrsam J Rössler F Horisberger K Hübel K Nilsson J de Rougemont O . Kidney retransplantation after graft failure: variables influencing long-term survival. J Transplant. (2022) 2022:3397751. doi: 10.1155/2022/3397751 35782455 PMC9242806

[23] Gritane K Jusinskis J Malcevs A Suhorukovs V Amerika D Puide I . Influence of pretransplant dialysis vintage on repeated kidney transplantation outcomes. Transplant Proc. (2018) 50:1249–57. doi: 10.1016/j.transproceed.2018.01.056 29880343

[24] Hariharan S Israni AK Danovitch G . Long-term survival after kidney transplantation. N Engl J Med. (2021) 385:729–43. doi: 10.1093/med/9780197697320.003.0001 34407344

[25] Freist M Bertrand D Bailly E Lambert C Rouzaire PO Lemal R . Management of immunosuppression after kidney transplant failure: effect on patient sensitization. Transplant Proc. (2021) 53:962–9. doi: 10.1016/j.transproceed.2020.10.009 33288310

[26] Lopez Del Moral Cuesta C Guiral Foz S Gomez Pereda D Perez Canga JL de Cos Gomez M Mazon Ruiz J . Immunosuppression with calcineurin inhibitor after renal transplant failure inhibits allosensitization. Biomedicines. (2020) 8:72. doi: 10.3390/biomedicines8040072 32231087 PMC7235765

[27] Yan L de Leur K Hendriks RW van der Laan LJW Shi Y Wang L . T follicular helper cells as a new target for immunosuppressive therapies. Front Immunol. (2017) 8:1510. doi: 10.3389/fimmu.2017.01510 29163552 PMC5681999

[28] Nimmo A McIntyre S Turner DM Henderson LK Battle RK . The impact of withdrawal of maintenance immunosuppression and graft nephrectomy on HLA sensitization and calculated chance of future transplant. Transplant Direct. (2018) 4:e409. doi: 10.1097/txd.0000000000000848 30584590 PMC6283087

[29] Khakhar AK Shahinian VB House AA Muirhead N Hollomby DJ Leckie SH . The impact of allograft nephrectomy on percent panel reactive antibody and clinical outcome. Transplant Proc. (2003) 35:862–3. doi: 10.1016/s0041-1345(02)04031-9 12644168

[30] Lachmann N Schonemann C El-Awar N Everly M Budde K Terasaki PI . Dynamics and epitope specificity of anti-human leukocyte antibodies following renal allograft nephrectomy. Nephrol Dial Transplant. (2016) 31:1351–9. doi: 10.1093/ndt/gfw041 27190369

[31] Schwab S Elmer A Sidler D Straumann L Stürzinger U Immer F . Selection bias in reporting of median waiting times in organ transplantation. JAMA Netw Open. (2024) 7:e2432415-e2432415. doi: 10.1001/jamanetworkopen.2024.32415 39254975 PMC11388028

[32] Woodside KJ Schirm ZW Noon KA Huml AM Padiyar A Sanchez EQ . Fever, infection, and rejection after kidney transplant failure. Transplantation. (2014) 97:648–53. doi: 10.1097/01.tp.0000437558.75574.9c 24637864

