# Supplementary figures and images for: The potential role of antimetabolite in preventing allosensitization before kidney retransplantation
Source: Front Immunol. 2026 Jul 8;17:1850824. doi: 10.3389/fimmu.2026.1850824 (PMC13388180; doi:10.3389/fimmu.2026.1850824)

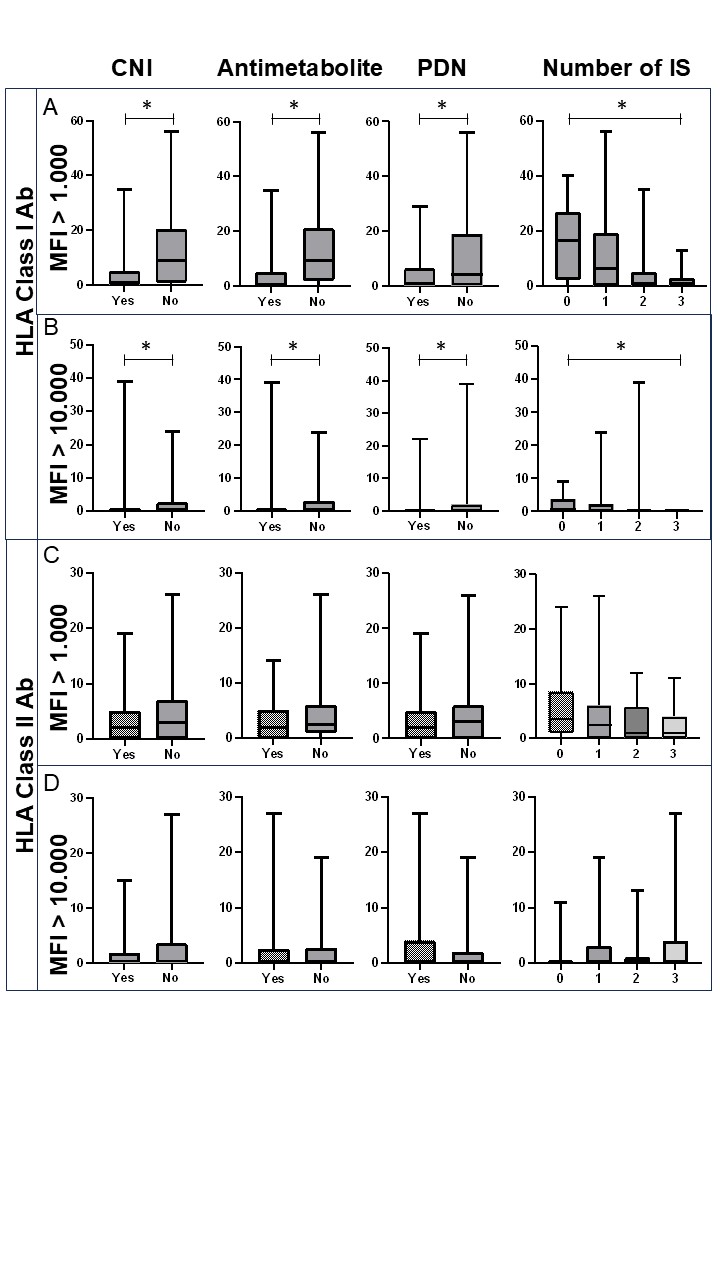

Supplement: Supplementary Figure 1 — CNI levels at the time point of failing graft and the day of retransplantation. [file Image1.jpeg]

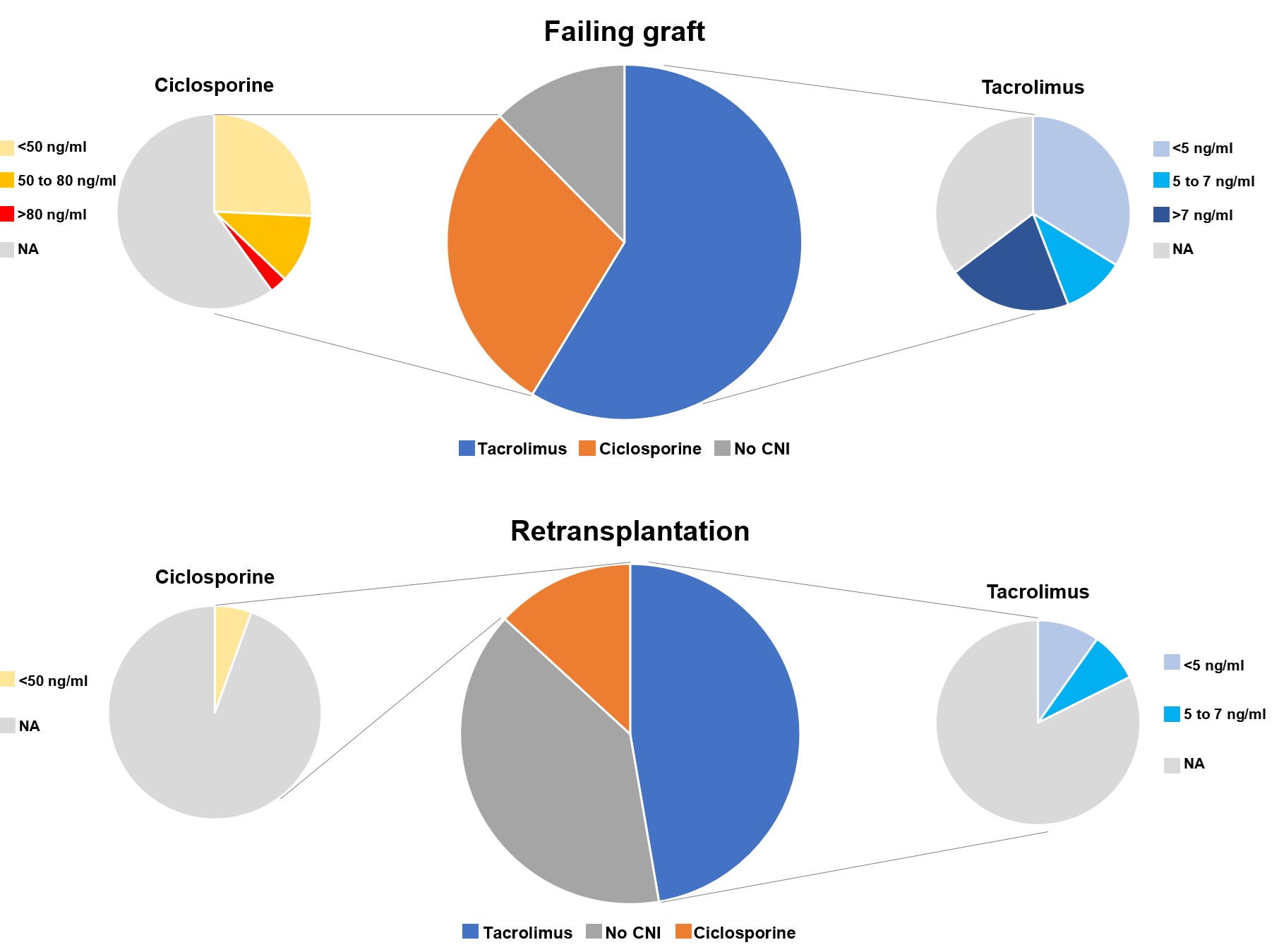

Supplement: Supplementary Figure 2 — Effect of immunosuppression on HLA antibodies. (A) Number of Class I Antibodies with MFI > 1,000 per patient at time of retransplantation. (B) Number of Class I Antibodies with MFI > 10,000 per patient at time of retransplantation. (C) Number of Class II Antibodies with MFI > 1,000 per patient at time of retransplantation. (D) Number of Class II Antibodies with MFI > 10,000 per patient at time of retransplantation. [file Image2.jpeg]
